# Carbolic Acid in the Treatment of Tetanus

**Published:** 1899-04

**Authors:** 


### THERAPEUTIC NOTES.

UNDER THE DIRECTION OF W. J. MARTIN, M.D.C.,
KANKAKEE, ILL.

CARBOLIC ACID IN THE TREATMENT OF TETANUS. A new treatment of tetanus has been advised by Baccelli, and consists of the subcutaneous injections of a solution of carbolic acid. An observation is furnished by Dr. Ziengo in which the injections were begun eight days after the first appearance of the first symptoms in a man having a complicated fracture of the forearm. A 3 per cent. solution in distilled water was used, and there was no sign of intolerance. The dose was raised to 50 centigrammes. No symptoms of poisoning were noticed with the exception of a slight degree of albuminuria. In all there was injected 978 centigrammes of carbolic acid in twenty-seven days, and, as Baccelli recommends, morphine (4 to 6 centigrammes) was given at first. The third day of treatment there was slight amelioration, and nine days later the tissues had disappeared. Complete recovery seems to have been obtained on the twenty-third day. This is the thirty-second case of tetanus treated by Baccelli's method, and there has been so far only one death, which is certainly very encouraging, as the ordinary mortality of tetanus is 70 per cent. Moreover, though a few cases have been cured by Dr. Roux's and Bond's treatment, a fresh series of cases terminated fatally; but this treatment seems to have been applied in desperate cases, therefore it is readily understood why the results are not more favorable.—*Therapeutic Gazette.*

